# Prevention of Occupational Hazards Due to Asbestos Exposure in Dentistry. A Proposal from a Panel of Experts

**DOI:** 10.3390/ijerph19063153

**Published:** 2022-03-08

**Authors:** Carmen Anaya-Aguilar, Manuel Bravo, Antonio Magan-Fernandez, Ramon del Castillo-Salmerón, Alberto Rodríguez-Archilla, Javier Montero, Eva Rosel, Paco Puche, Rosa Anaya-Aguilar

**Affiliations:** 1 Department of Economy and Business Administration, University of Málaga, Campus El Ejido, 29071 Málaga, Spain; canaya@uma.es (C.A.-A.); ranaya@uma.es (R.A.-A.); 2 Department of Preventive and Community Dentistry, Faculty of Dentistry, University of Granada, Campus de Cartuja s/n, 18071 Granada, Spain; mbravo@ugr.es; 3 Department of Periodontics, Faculty of Dentistry, University of Granada, Campus de Cartuja s/n, 18071 Granada, Spain; 4 Department of Prosthodontics, Faculty of Dentistry, University of Granada, Campus de Cartuja s/n, 18071 Granada, Spain; rdcastil@ugr.es (R.d.C.-S.); erosel@ugr.es (E.R.); 5 Department of Oral Medicine, Faculty of Dentistry, University of Granada, Campus de Cartuja s/n, 18071 Granada, Spain; alberodr@ugr.es; 6 Department of Surgery, Faculty of Medicine, University of Salamanca, 37007 Salamanca, Spain; javimont@usal.es; 7 OTRI, University of Malaga, Campus El Ejido, s/n, 29071 Málaga, Spain

**Keywords:** asbestos, chrysotile, mesothelioma, dental technician

## Abstract

Asbestos in all its forms is a Group 1 material agent with proven carcinogenic effects in the human being since 1977. Exposure to asbestos can be considered unsafe. The use of asbestos in the field of dentistry had a common use in the manufacture of dental prostheses in the 1960s and 1970s. Taking into account the long induction period of this agent and the plausibility for being a risk factor in dentistry, the objective of this study is to propose a plan for the prevention of occupational risks due to asbestos exposure in dentistry by means of the contribution of a panel of experts. An Expert Panel (EP) approach was used in which a group of nine experts identified and documented the use of asbestos in the dental profession. EP was created and followed the protocol in accordance with the EuropeAid Assessment Guidelines. As a result of this study, EP documented the common use and sources of asbestos in dentistry in prosthetic materials, dental dressings, and in the coating of casting cylinders. EP also created a consensus document on the priority measures for the Plan for the Prevention of Risks from Asbestos in Dentistry, based on previous reports from the European Commission Senior Labour Inspectors’ Committee. The document concluded that obtainment of information, receiving specific training on the subject and performing epidemiological studies, and the proper risk assessments were the priority measures to adopt.

## 1. Introduction

Asbestos is a commercial term used to identify the fibrous-asbestiform variety of six minerals: chrysotile (serpentine asbestos) and five amphiboles, i.e., actinolite asbestos, amosite (asbestos cummingtonite-grunerite), anthophyl-lite asbestos, crocidolite (asbestos riebeckite), and tremolite asbestos [1]. Etymologically, asbestos means pure, incorruptible, and is from the Latin word amiantus, and this from the Greek word ἀμίαντος (/amíantos/), which literally means “without stain” [2].

Among this group of silicates, two mineralogical groups are of special interest: serpentines and amphiboles. Both are metamorphic rocks that crystallize in fibrous ways [3]. Among the serpentines is the chrysotile, for which its trade name is white asbestos, which has an industrial use of 90 to 95%. Amphiboles include crocidolite, a variety commercially known as blue asbestos, which has an industrial use of 5–7%, and amosite or brown asbestos, for which its industrial use represents 3–5% [3]. Serpentine fibres are flexible and curved, and amphibole fibres are generally straight, brittle, and often needle-shaped. These fibres are formed by bundles of parallel fibrils and are easily separable (friable). The elementary fibrils are imperceptible; cannot be seen, smelled, touched, tasted, or tasted; and the only way to visualize them is through microscopy [3].

Asbestos in all its forms according to the International Agency for Research on Cancer (IARC) is considered as a Group 1 type “substance carcinogenic to humans” [1], and according to the National Institute of Occupational Health and Safety (INSH) of Spain, “no exposure to asbestos, however small, can be considered safe” [3]. Its use dates back some 3000 years ago, according to archaeological samples, in the area of present-day Finland; there is also evidence of its use in ancient Egypt in the clothes used to embalm Pharaohs and it was the Greeks, some 2000 years ago, who coined the term “asbestos”, which means inextinguishable. Strabo in the 1st century AD noted that slaves in contact with asbestos suffered from lung disease and Pliny the Elder observed that people who had been in contact with asbestos dust were more likely to die at an early age. The use of asbestos did not become popular until the Industrial Revolution and was used willy nilly in the First and Second World Wars [4].

In 1955, a report by Richard Doll showed that lung cancer was a specific industrial hazard found between certain asbestos British workers. The average risk for lung cancer among men employed for 20 or more years was 10-times higher compared to the one observed in general population. Moreover, this risk became progressively lower as the employment time in hazardous conditions decreased [5].

In the 1960s–1970s, it was known that asbestos was a health hazard; already in 1964, Dr Irving Selikoff published a study proving that people who worked with asbestos-containing materials had a high incidence of asbestosis, lung cancer, and mesothelioma [6]. However, Stanton et al. reported in 1981, in a study performed in rodents, that carcinogenicity of asbestos fibers depends on several factors, such as the size and durability rather than other previously thought physicochemical properties of these materials, emphasizing the idea that all respirable asbestos fibers should be viewed as hazardous [7]. Despite this knowledge, many countries, as in the case of Spain, recorded the maximum use of asbestos in industry, construction, and even in domestic use during this period [4]. Wagner et al. (1960) reported cases of this disease in the population near asbestos extraction mines in South Africa [8], and research such as those of Maule et al. (2007) [9] and Tarrés et al. (2013) [10] analysed the relationship between environmental exposure and the risk of mesothelioma.

Asbestos-related diseases are caused by exposure to these asbestos fibers, which may be benign and of a malignant type. Nonmalignant asbestos diseases include asbestosis, COPD, pleural plaques, pleural thickening, pleural effusion, and atelectasis. According to monograph 100c by the IARC, several studies using animal models have evaluated the carcinogenic potential of asbestos fibers from 1970 to 2000s and were summarized in this publication, concluding that that there is enough scientific evidence to confirm that all asbestos species can cause several malignant disorders [11]. Malignant asbestos diseases include mesothelioma, lung cancer, ovarian cancer, and laryngeal cancer [12]. The main pathogenic mechanism of asbestos-related injury is interstitial fibrosis caused by deposition and transmigration of asbestos fibers in the lung tissue. This deposition generates an accumulation of macrophages and fibroblasts that result in fibrosis. Reactive oxygen species contribute to a chronic inflammatory state. These reactive oxygen species and transepithelial migration of fibers cause damage to type 1 alveolar cells. Other inflammatory mediators are also involved in this process, such as tissue necrosis factors, interleukins, and stimulation of the phospholipase C pathway.

In Spain, its use and commercialization are prohibited by the Ministerial Order of 7 December 2001, which became fully effective in 2002 and which incorporates European Directive 1999/77/EC into Spanish domestic law, limiting the marketing and use of certain dangerous substances and preparations (asbestos). However, it still permits asbestos that is already installed or “in service” before the date of its entry into force and until its elimination or the end of its useful life [13].

According to Bernardo and Puche [3], there are no reliable records of asbestos victims apart from the ones provided by Tossavainen in 2004, in which a relationship between asbestos consumption and mesothelioma-related deaths is established, with a multiplier factor of 3.8, i.e., each death from mesothelioma is equivalent to a total of 3.8 total victims [14].

Previous reports related the deaths caused by mesothelioma [3,15], which Tossavainen previously set in 2004 at one death caused by mesothelioma for every 130 tons of asbestos consumed [14], by considering that the data of asbestos consumed in the twentieth century had been accounted as decennial periods [16]. Thus, the model built for measuring this association has estimated more than 5 million deaths worldwide by exposure to asbestos. In Spain, an average of 112,000 deaths has been accounted for by the use of asbestos during the twentieth century, and it has been estimated that about 40% of them has yet to come. The current mortality in Spain related to mesothelioma in the last 20 years, according to official data from the Spanish National Statistics Institute, is 6983 [17].

Concerning the dental sector, we find, in the scientific literature, the work of a group of Italian experts indicating that the use of asbestos was common in the manufacture of dental prostheses in the 1960s and 1970s. According to the results of their study, past exposure to materials previously used in the manufacture of dental prostheses could trigger the development of malignant mesothelioma of the pleura, as other kinds of exposures to asbestos-containing materials also do [18].

Asbestos was used for the manufacture of several dental products, such as a binder in dental dressings and as a lining material for casting cylinders [19]. Italian researchers, experts in environmental science and occupational health, conducted an analysis of over 5000 patients with pleural mesothelioma between 2000 and 2014, finding four subjects whose only exposure to asbestos had been their work as dental technicians [18]. The inner lining of the casting cylinder contained asbestos from the 1930s to at least the 1970s and was used in the lost wax method of casting crowns, bridges, and other metal prosthetic devices [20], and the 1988 Spanish edition of the Dental Laboratory Procedure Manual Volume II Fixed Prosthodontics [21] contains a thorough explanation of its use and how to apply the asbestos material.

Ferrer and Martínez (2008) [22] predicted an increase in cases in Argentina until the middle of the 21st century, despite the fact that asbestos fibres have been banned since 2003 due to the long latency period. Those affected by the inhalation of asbestos fibres can be asymptomatic for a period between 35 and 40 years. Once symptoms appear, life expectancy is very short and the quality of life deteriorates rapidly. On this basis, the concern raised is that many professionals and perhaps patients were unconsciously exposed to these fibres and may now be approaching the time when the manifestation of occupational disease could be common in any branch of the dental profession.

Bakdash and Frydman (1976) [23] explored the potential threat of the presence of asbestos in dental dressings and examined several products available in America, revealing that The Periodontal Pack Corp of America produced periodontal dressings with powders containing 14.29% asbestos by weight. In April 1976, the American Dental Association took the decision to no longer accept these products, even though they made a statement that they did not pose a danger to patients. Dyer [24] wrote in 1967 about the adverse effects of asbestos and periodontal dressings and explained that mixing asbestos with the patient could expose both the dentist and the patient to harmful doses of this product. Reid et al. (1991) [25] stated that, in addition to the use of asbestos in periodontal dressings, there was also significant exposure to asbestos fibres in the denture manufacturing process. Their research explains that, in 1971, one of the authors of the report saw mesothelioma in a dental technician and assumed that it may have been caused by dust from dry asbestos papers, which could pose a threat to the health of dental technicians and dentists.

According to the 1991 report published in Lancet [25], it was common in undergraduate dental student practices to use a minimum of 40 sets of dentures, and it is possible that those using these rolls of paper may have been exposed to asbestos and if the period for symptoms to materialize is 35 to 40 years, it follows then that many dentists, technicians, and dental assistants now manifest some form of disease related to it. Asbestos is banned in more than 55 countries but is still allowed in the remaining ones, mainly in emerging countries that represent a population of more than 70% of the world’s total [3]. The result of this research is alarming and should be of great concern to dental professionals and their families.

As concluded by Fry et al. in 2009, there is a raising concern that there may be many dental sector professionals who have been unconsciously exposed to high levels of asbestos-containing materials [26]. These professionals are currently reaching certain ages at which these forms of signs and symptoms may be more prevalent and could be a consequence of unknown previous exposure. Therefore, there is enough reason to consider this exposure as an occupational risk factor in dentistry, especially if we take into account the long induction period of the diseases caused by this exposure. To date, no specific prevention program for this risk factor and exposure in dentistry has been published, neither at the national nor international levels. Therefore, the objective of this study is to propose a plan for the prevention of occupational risks due to asbestos exposure in dentistry by means of the contribution of a panel of experts.

## 2. Materials and Methods

In the third quarter of 2020, we asked a panel of experts (EP) to identify the key elements in an occupational risk prevention plan for asbestosis in dentistry. The EP was formed and worked according to the EuropeAid Assessment Guidelines [27]. Briefly, an EP is a research technique in which a group of experts on the topic under study meet and reach conclusions and recommendations by consensus, based on a precise and replicable work plan. All the experts met the following criteria:

‑ Experience, ability, and professional recognition by the other panellists;
‑ Independence from evaluation;
‑ Teamwork skills.

In the first stage, EP divided the work between three pairs of experts to address the issue from three perspectives: one trio dealt with asbestos in dentistry, another dealt with different proposed plans for prevention, and a third dealt with asbestos as a general public health problem.

Thus, they developed an initial draft. This draft was discussed in depth until a final consensus was reached on the objectives (for variables and targets), ensuring compliance with SMART conditions: Specific, Measurable, Achievable, Realistic, and Time-bound [27].

Based on the European Commission’s “Non-binding guide to good practice to prevent or minimise the risks of asbestos in work where it is present (or likely to be present) for employers, workers and labour inspectors published by the Senior Labour Inspectors Committee (SLIC)” [28], the EP was tasked with defining the items that are of interest to dentistry and extracted a total of 24 variables. The trios then prioritized which preventive measures they would propose, and from a minimum of 4 proposals, the 24 most important ideas were selected.

In order to prioritize in a simple manner through consensus, each voter was asked by email to indicate, from the 24 ideas pre-selected by the trio, 10 ideas that according to their criteria are the most relevant and that allowed each voter to indicate the one marked by at least five experts as a priority idea.

In a second stage, the number of experts who supported each measure was added up, which made it possible to classify the measures as more or less relevant. Measures with a high level of consensus were considered to be very relevant.

The EP workplan was scheduled as follows:

✽ November 2020: Participation proposal to all potential EP members and electronic exchange of scientific data;
✽ January–February 2021: Online meeting of the EP members to define the best method, define the objectives of an asbestos exposure prevention plan in dentistry, schedule of the workplan, assignment of duties of the members, and work chronology;
✽ April 2021: A draft was prepared and presented at the meeting with 9 participants;
✽ May 2021: Second telematic meeting of the EP to discuss the draft and amendments;
✽ June 2021: EP e-discussion on the outcome document;
✽ July 2021: acceptance of the final version by EP members.

In the Faculty of Dentistry of Granada and in its museum, EP identified and photographed the techniques and elements that could have used asbestos.

The EP proposed an occupational risk prevention plan for asbestos exposure in dentistry for which its variables were selected following the “Non-binding guide to good practice to prevent or minimise the risks of asbestos published by the Senior Labour Inspectors Committee (SLIC)” published by the European Commission [28].

## 3. Results

**Figure 1:**
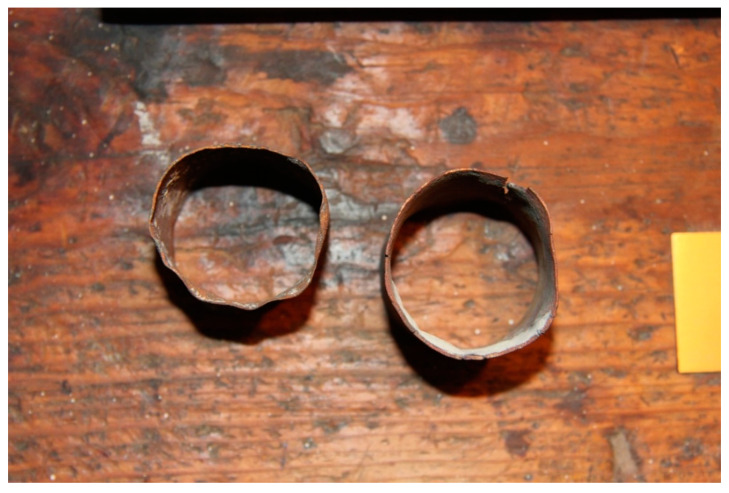
Casting Cylinders. Source: Dental Museum. Faculty of Dentistry. University of Granada (2021).

**Figure 2:**
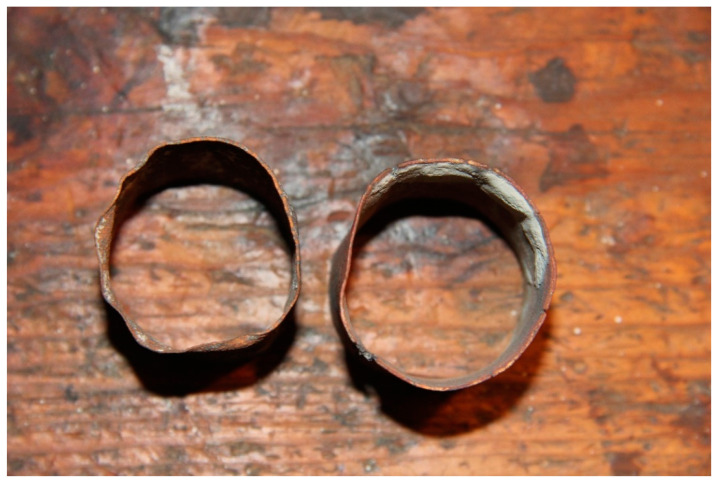
Asbestos liner inside casting cylinder. Source: Dental Museum. Faculty of Dentistry. University of Granada (2021).

**Figure 3:**
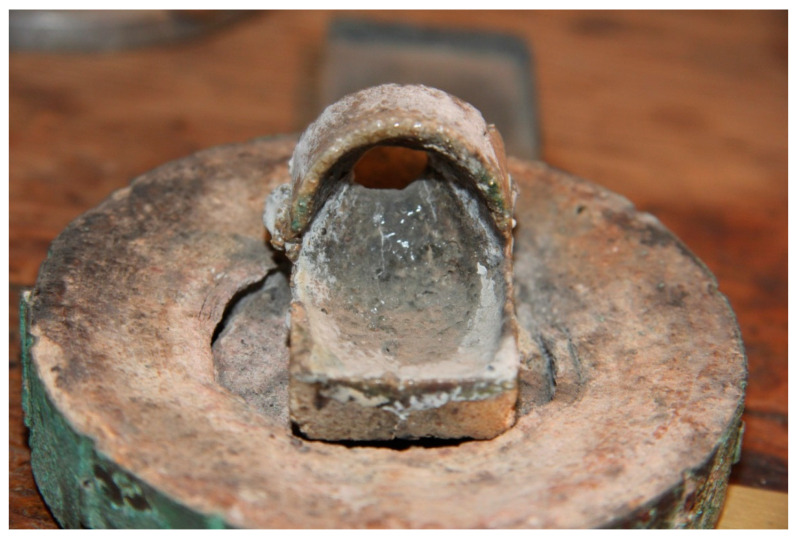
Crucible for melting metals that would be later inserted into a Solbrig-Platshick casting cylinder. Source: Dental Museum. Faculty of Dentistry. University of Granada (2021).

At the Faculty of Dentistry of the University of Granada, EP identified the instruments used that could have contained asbestos and basically located them in the casting cylinders, as shown in Figure 1, and in the liners used to form the wax pattern (Figure 2). Figure 1 shows the casting cylinders used to form the wax pattern with a resilient coating on the inside to prevent distortion of the casting by counteracting the shrinkage of both the metal and the wax. Figure 2 shows part of this coating on the casting cylinder. In the final discussion of the paper, EP concludes that the dental professional may have been exposed to asbestos material at various stages of handling the instrument and casting, such as the investment or placement of the asbestos strips on the cylinders; removal of the instrument in the cylinder; hydration of the dry asbestos; hand or Vac-U- Spat; in the removal of the cylinder and its base from the Vac-U-Spat lid; in the separation of the Vac-U-Spat shaft from the vibrating button; in the asbestos lining of the crucible (Figure 3); in the heating of the crucible with an air–gas torch to remove impurities from the asbestos lining; in the cleaning of the casting; in the finishing and polishing operations; and in the disposal in garbage.

Moving forward in the results, EP proposed the elaboration of an Occupational Risk Prevention Plan for Asbestos in Dentistry, the prioritized measures of which are depicted in Table 1.

The EP had full consensus on the need to “Have the necessary information to know whether or not materials contain asbestos” and “Know what measures to take if you encounter asbestos-containing materials”. Other measures that are close to total consensus are “Know how to recognize products that may contain asbestos” and the need to “Carry out specific epidemiological studies in the dental profession in relation to asbestos”, “Use appropriate protective equipment”, “Have a risk assessment and a specific written work plan”, “Organize training sessions with competent experts in the field”, and “In case of exposure to asbestos, evaluate whether medical surveillance is recommended or necessary” (Table 1).

## 4. Discussion

The EP recognizes, with respect to the instruments kept in the museum of the Faculty of Dentistry of Granada, that the dental professional could have been exposed to asbestos material in the techniques of casting and investment of wax patterns (as already published by Reid in 1991) [25], for which the three basic steps for the realization of a casting are as follows: investment of the castable wax pattern to duplicate the anatomical features and melting the wax to leave a negative on which the molten alloy will be introduced in liquid state (casting) [29].

For many years, the material used to coat these cylinders was asbestos due to its mechanical properties, heat resistance, and incombustibility. Although this product is currently withdrawn from the market due to its carcinogenic properties, its substitute, ceramic paper, has also been reported to be as dangerous as asbestos, because it contains fibres of a size that also produce lung cancer [29,30,31,32]. In the cylinder, a dry strip of asbestos was glued by carefully adapting it to the internal face of the cylinder, a strip that when peeled off dispersed the particles of this material. The main function of this material in the liners was to absorb the expansion of the liner inside the metal cylinder. Without it, this expansion would occur inwards, distorting the mould and, therefore, the casting. In addition, the asbestos allowed the liner to be more easily removed from the cylinder.

EP considers as a priority the need to transmit information on the potential risks for dental professionals of having been exposed to asbestos (Table 1) in accordance with the evidence in the scientific literature [23] and epidemiological data such as the incidence of Mesothelioma in Spain, which continues to grow in recent years [17], even though the prohibition of its use and commercialization in Spain, through the Ministerial Order of 7 December 2001, became fully effective in 2002. However, this regulation allows asbestos that is already installed or “in use” before the date the Regulation to be maintained until its elimination or the end of its useful life [13]. Since 2008, training programs such as those for dental technician assistants in Spain include practices with asbestos [33] and, therefore, contribute to the possibility that asbestos could be used by professionals of the dental sector in places where asbestos use is not forbidden. From a preventive point of view, it is reasonable to assume that, even years after its prohibition, these professional practices could have continued or still be common in certain permitted locations; therefore, the dental sector should include asbestos in their risk assessments.

The trace of asbestos-containing materials is an occupational health problem and an area with specific regulations. Similarly, asbestos is considered to be a major environmental pollutant and public health problem due to its widespread presence in almost all activities in view of its dangerousness [34]. It is also noted that, in the current regulations, Royal Decree 396/2006 [35] does not specifically include the dental sector; thus, EP concludes that there is a need to establish guidelines that allow the recognition of materials that could contain asbestos, to know the procedure if they contain it, to carry out epidemiological studies on the topic, and to carry out periodic risk assessments as indicated in Table 1, following the recommendations of the Committee of Senior Labour Inspectors of the Labour Inspectorate [28].

It is observed in the literature used in this work that, in many industrialized countries, there is a decrease in asbestos consumption from the 1990s due to the accumulated knowledge of health risks [3,22,36]; moreover, given the prolonged latency period of this effects, an increase in cases is expected in the following years [17]. These arguments reinforce the EP consensus on a specific medical surveillance system (Table 1) that also avoids the underestimation of health problems arising from asbestos with other diseases such as smoking [37].

A recent review by Ierardi et al. (2021), it describes the potential asbestos exposure of dental professionals in the USA. The authors conclude that although specific exposure is possible, measurements do not exceed regulatory standards; therefore, they do not expect the use of dental tapes and liners to pose an increased health risk in these professionals. To support their conclusion, they stated that epidemiological studies do not report increased risk in this sector. However, in the limitations of their study, they also report that exposure data is scarce and that they assume a low exposure by basis [38]. These conclusions support the aim of our work, which is to confirm the presence of asbestos exposure in dental professionals first and the need to establish adequate prevention plans accordingly.

Since the problem of asbestos exposure goes beyond the dental sector, further studies should also include experts on its mineralogical nature, its toxic and pathogenic properties, and biological responses to its exposure as well as epidemiological studies. All of these would provide a multidisciplinary and comprehensive approach to the topic in further research lines.

## 5. Conclusions

This study documented the common use of asbestos in the dental profession both in prosthetic materials, dental dressings, and in the lining of casting cylinders so to highlight the fact that dental professionals may have been exposed to the harmful effects of this material.

Although asbestos is banned in many countries, there are others where it is permitted. The panel of experts in the development of this research has been able to access video recordings of prosthetic practices that include asbestos as a material.

It is necessary to inform and train the profession of the possible risk of having been exposed to asbestos fibres, as well as the implementation of a prevention plan for which its starting point would include adequate epidemiological studies and an adequate risk assessment and the inclusion of the dental sector in the lists of professions with exposure to asbestos. Authors should discuss the results and how they can be interpreted from the perspective of previous studies and of the working hypotheses. The findings and their implications should be discussed in the broadest context possible. Future research directions may also be highlighted.

## Figures and Tables

**Table 1:** Preventive measures proposed by the expert panel.

Item	Preventive Measures Proposed by Experts	Total Points
1	Have the necessary information to know whether or not the materials contain asbestos, the type of asbestos, and if it could be airborne.	9
2	Knowing what to perform if you encounter asbestos-containing materials	9
3	Knowing how to recognize products that may contain asbestos	8
4	Conduct specific epidemiological studies in the dental profession in relation to asbestos.	8
5	Use appropriate protective equipment	7
6	Have a specific written risk assessment and work plan in place	6
7	Organizing training sessions with relevant experts in the field	6
8	In case of asbestos exposure assess whether medical surveillance is recommended or necessary.	6
9	In case of probability of having had contact with asbestos, have medical supervision.	5
10	Include the dental profession in the tables of professions with asbestos exposures.	5
11	Ensure that workers are trained in the function, choice, and use of protective equipment.	4
12	In case of disposal of asbestos-containing waste follow best practice according to national and international standards.	3

## Data Availability

All the results from the study are available in the present manuscript. Any further data will be provided at request to the authors of the manuscript.

## References

[1] IARC (1973). Some Inorganic and Organometallic Compounds.

[2] Real Academia Española (2021). Diccionario de la Lengua Española.

[3] Bernardo A., Puche P. (2019). Todo Sobre el Amianto. Una Guía Visual.

[4] MAPFRE (2008). Análisis Retrospectivo de la Exposición de Trabajadores del Sector de la Construcción Naval al Amianto y de su Relación Causa-efecto Con Patologías del Aparato Respiratorio.

[5] Doll R. (1993). Mortality from lung cancer in asbestos workers 1955. Br. J. Ind. Med..

[6] Selikoff I.J., Nicholson W.J., Langer A.M. (1972). Asbestos air pollution. Arch. Environ. Health.

[7] Stanton M.F., Layard M., Tegeris A., Miller E., May M., Morgan E., Smith A. (1981). Relation of particle dimension to carcinogenicity in amphibole asbestoses and other fibrous minerals. J. Natl. Cancer Inst..

[8] Wagner J.C., Sleggs C.A., Marchand P. (1960). Diffuse pleural mesothelioma and asbestos exposure in the north western cape province. Br. J. Ind. Med..

[9] Maule M.M., Magnani C., Dalmasso P., Mirabelli D., Merletti F., Biggeri A. (2007). Modeling mesothelioma risk associated with environmental asbestos exposure. Environ. Health Perspect..

[10] Tarres J., Alberti C., Martinez-Artes X., Abos-Herrandiz R., Rosell-Murphy M., Garcia-Allas I., Krier I., Cantarell G., Gallego M., Canela-Soler J. (2013). Pleural mesothelioma in relation to meteorological conditions and residential distance from an industrial source of asbestos. Occup. Environ. Med..

[11] IARC (2012). Arsenic, Metals, Fibres, and Dusts.

[12] Mossman B.T., Gee J.B. (1989). Asbestos-related diseases. N. Engl. J. Med..

[13] European Parliament (2003). Directive 2003/18/EC of the European Parliament and of the Council of 27 March 2003 amending Council Directive 83/477/EEC on the Protection of Workers from the Risks Related to Exposure to Asbestos at Work.

[14] Tossavainen A. (2004). Global use of asbestos and the incidence of mesothelioma. Int. J. Occup. Environ. Health.

[15] Puche P. (2017). Amianto. Una Epidemia Oculta e Impune.

[16] Virta R.L. (2006). Worldwide Asbestos Supply and Consumption Trends from 1900 through 2003.

[17] National Institute of Statistics of Spain (2020). Deaths According to Causes (Chapters) by Sex and Age Group.

[18] Mensi C., Ciullo F., Barbieri G.P., Riboldi L., Somigliana A., Rasperini G., Pesatori A.C., Consonni D. (2017). Pleural malignant mesothelioma in dental laboratory technicians: A case series. Am. J. Ind. Med..

[19] Cook A. (2008). Asbestos dressing. Br. Dent. J..

[20] Markowitz S.B., Moline J.M. (2017). Malignant mesothelioma due to asbestos exposure in dental tape. Am. J. Ind. Med..

[21] Rhoads J.E., Rudd K.D., Morrow R.M. (1988). Procedimientos en el Laboratorio Dental. Tomo ii. Prótesis Fija.

[22] Ferrer J., Martínez C. (2008). El diagnóstico de las enfermedades respiratorias causadas por el asbesto. Arch. Bronconeumol..

[23] Bakdash M.B., Frydman A. (1976). Asbestos in periodontal dressings: A possible health hazard. Quintessence Int. Dent. Dig..

[24] Dyer M.R. (1967). The possible adverse effects of asbestos in gingivectomy packs. Br. Dent. J..

[25] Reid A.S., Causton B.E., Jones J.S., Ellis I.O. (1991). Malignant mesothelioma after exposure to asbestos in dental practice. Lancet.

[26] Fry C. (2009). An investigation into asbestos related disease in the dental industry. Br. Dent. J..

[27] European Commission (2007). Summary of Expert Panel.

[28] Senior Labour Inspectors Committee (2021). A Practical Guide on Best Practice to Prevent or Minimise Asbestos Risks in Work That Involves (or May Involve) Asbestos: For the Employer, the Workers and the Labour Inspector.

[29] Shillingburg H.T.J., Hobo S., Whitsett L.D., Jacobi R., Brackett S.E. (2011). Fundamentos Esenciales en Prótesis.

[30] Priest G., Horner J.A. (1980). Fibrous ceramic aluminum silicate as an alternative to asbestos liners. J. Prosthet. Dent..

[31] Davis D.R. (1987). Potential health hazards of ceramic ring lining material. J. Prosthet. Dent..

[32] Naylor W.P., Moore B.K., Phillips R.W. (1987). A topographical assessment of casting ring liners using scanning electron microscopy (sem). Quintessence Dent. Technol..

[33] Public Service of Employment (2008). Auxilliary Dental Technician. Training Program.

[34] Comisión de Seguridad y Salud Laboral del Consejo de Relaciones Laborales de Cataluña (2018). Exposición Laboral a Fibras de Amianto en Cataluña.

[35] Estado B.O.D., Real decreto 396/86/2006 (2006). De 31 de Marzo, por el que se Establecen las Disposiciones Mínimas de Seguridad y Salud Aplicables a los Trabajos con Riesgo de Exposición al Amianto.

[36] International Social Security Association (2019). Asbestos. Towards a Worldwide Ban.

[37] Furuya S., Chimed-Ochir O., Takahashi K., David A., Takala J. (2018). Global asbestos disaster. Int. J. Environ. Res. Public Health.

[38] Ierardi A.M., Mathis C., Urban A., Jacobs N., Finley B., Gaffney S. (2021). Potential airborne asbestos exposures in dentistry: A comprehensive review and risk assessment. Crit. Rev. Toxicol..

